# Supplementary material for: Association of Progranulin Gene Expression from Dyspeptic Patients with Virulent Helicobacter pylori Strains; In Vivo Model
Source: Microorganisms. 2022 May 10;10(5):998. doi: 10.3390/microorganisms10050998 (PMC9145319; doi:10.3390/microorganisms10050998)
Supplement: Supplementary file 1 [file microorganisms-10-00998-s001.zip › microorganisms-1655161-supplementary.pdf]

Supplementary Materials:

S1. Primers for PCR assays

Table S1. Primers used for confirmation of species and study of virulence of *Helicobacter pylori*

Gene	Primers 5′- 3′	Size (bp)	mT ºC	References
RNA 16S 27 F	AGAGTTTGATCCTGGCTCAG	1350	53.0	[26]
RNA 16S 1392 R	GGTTACCTTGTTACGACTT			
ureC F	AGCTATAAAGTCGGCGAGAG	224	57.0	[27]
ureC R	ATTGCACCCGTTAGGCTCAT			
vacA s1/s2 F	ACAACAAACACACCGCAAAA	s1=249 s2=276	54.2	*
vacA s1/s2 R	GCTTGAATGCGCCAAACT			
vacA m1/m2 F	CAATCTGTCCAATCACGAG	m1=567	53.1	[2]
vacA m1/m2 R	GCGTCAAAATAATTCCAAGG	m2=642		
iceA 1 F	GGGCGATTGAATATGATGGT	225	52.5	*
iceA 1 R	GCAACCGCATGGATATTTTC			
iceA 2 F	CGGGTTGTTTTGAGGGTAAA	313	53.2	*
iceA 2 R	AGCAACAATATGCCCATCAG			
cagA F	CAGTGGCTAAAGCAATGGGC	1119	56.0	*
cagA R	CATTTTCCCTCGCCAAGCAG			

* Primers designed exclusively for the study in the MEGA 6 program, validated in the NCBI database and manufactured by IDT (Integrated DNA Technologies, USA).

S2. Virulence *H. pylori* characterization

Table S2. Characterization of the virulence of *Helicobacter pylori* strains, including *s* and *m* alleles of *vacA* and *A1* and *A2* of *iceA* alleles, colonizing in the different gastric epithelium status.

Virulence genes	Total n=75	NL n=46	L n=29	p Value	Injuries		
					NEL n=11	EL n=14	AtL n=3
vacA	75 (100)	46 (100)	29 (100)	0.407	11 (100)	14 (100)	3 (100)
s1m1	23 (30.67)	12 (26.09)	11 (37.93)	0.369	3 (27.27)	5 (35.72)	2 (66.67)
s1m2	7 (9.33)	3 (6.52)	4 (13.79)	0.108	3 (27.27)	1 (7.14)	
s2m1	8 (10.67)	5 (10.87)	3 (10.35)	0.980	1 (9.09)	2 (14.28)	
s2m2	29 (38.67)	19 (41.30)	10 (34.48)	0.971	4 (36.36)	5 (35.72)	1 (33.33)
m indet	3 (4.00)	2 (4.35)	1 (3.45)	>0.999		1 (7.14)	
s indet	5 (6.67)	5 (10.87)					
iceA	38 (50.67)	19 (41.30)	19 (65.52)	0.340	7 (63.64)	8 (57.14)	3 (100)
A1	14 (36.84)	8 (42.10)	6 (31.58)	0.947	2 (28.57)	2 (25.00)	1 (33.33)
A2	20 (52.63)	8 (42.10)	12 (63.15)	0.197	5 (71.43)	5 (62.50)	2 (66.67)
A1/A2	4 (10.52)	3 (15.80)	1 (5.27)	>0.999		1 (12.5)	
cagA	34 (45.33)	20 (43.48)	14 (48.27)	> 0.999	3 (27.27)	8 (57.14)	2 (66.67)

NL= Non lesion; L= Lesion; NEL= Non-erosive lesion; EL= Erosive lesion; AtL= Atrophic lesion; Comparison between variables according to the Chi-square test. Virulence genes determined by DNA amplification for *vacA*, *cagA* and *iceA* genes by Polymerase Chain Reaction PCR assay, and PCR-RFLP polymorphic restriction assay for confirmation of vacA *s*, *m* alleles, visualized in gel agarose (Cleaver Scientific. Rugby, UK). The status of the gastric tissue was determined according to the report of the endoscopic observation. Statistical significance $p<0.05$.

S3. Mainly virulence profiles of *H. pylori* strains.

Observing the combinations of these genes (Figure S1), it can be seen that in NL tissues, the strains that express only the *vacA* gene predominated (n=17, 36.96%), condition that is modified in EL, where the profile that brings together the three *vacA, cagA, iceA* genes, detected, predominates in five isolates (35.71%), but not in NEL, in this group $VacA^{+}IceA^{+}$ strains predominated or only $VacA^{+}$ present in the same number of biopsies (n=4, 36.36% for each one, respectively), no found $VacA^{+}CagA^{+}$ isolates and two isolates (66.67%) presented the three *vacA, cagA, iceA* genes and *vacAs1m1* and i*ceA1* alleles, this profile is also predominant in AtL, in this group, three biopsies were studied, two colonized by $VaAs1m1^{+}CagA^{+}IceA1^{+}$ strains (n=2, 66.67%).

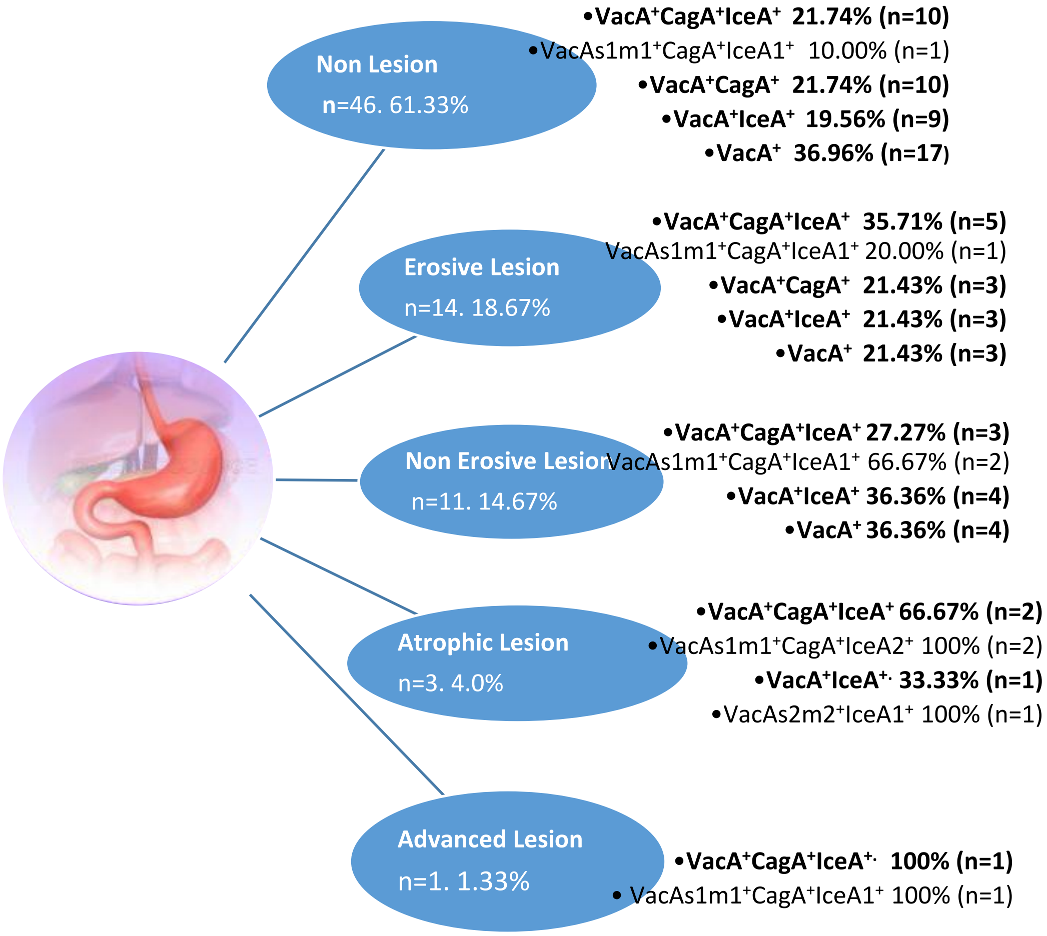

Figure S1. Distribution of *vacA, cagA, iceA* genes in *H. pylori* isolates obtained from gastric biopsies.

n= number of *H. pylori* strains. %= Distribution in percentage of the averages. Virulence genes determined by DNA amplification for *vacA, cagA* and *iceA* genes by PCR method, and PCR-RFLP polymorphic restriction assay for confirmation of vacA s and m alleles visualized on agarose gel (Cleaver Scientific. Rugby, UK). The gastric tissue status was determined according to the report of the endoscopic observation. Statistical significance $p<0.05$.

S4. Ethical Aspects

S 4.1. Informed consent.

Approved by the Scientific Ethics Committee of the Universidad de La Frontera, Chile (Protocol no. 028/18).

CONSENTIMIENTO INFORMADO

Proyectos: Evaluación del perfil de virulencia y resistencia a antibióticos en *Helicobacter pylori* como determinantes de la severidad de lesiones gástricas y eventual falla terapéutica (FONIS SA16I0197).

Asociación de la virulencia de cepas de Helicobacter pylori con la severidad de las lesiones gástricas en modelos in vivo e in vitro. Papel de la progranulina en la evolución de las lesiones (CONICYT Nº 21171513).

Financiamiento: Fondo Nacional de Investigación y Desarrollo en salud – Beca Doctorado Nacional CONICYT.

El presente documento contiene preguntas y respuestas respecto al estudio del cual ha sido invitado a participar. Consulte cualquier duda que tenga al respecto, en el momento de aceptar participar o cuando estime conveniente acercándose a alguno de los investigadores que participarán del estudio. Este documento será presentado y explicado a usted por el personal perteneciente al estudio, en el Hospital Hernán Henríquez Aravena o en la Clínica Alemana de Temuco.

Usted es invitado a participar en este estudio, porque su doctor le indicó una endoscopia digestiva alta debido a los síntomas digestivos que presenta, como dolor, acidez, malestar; o bien por tener familiares con historia de enfermedades gástricas.

1. ¿Cuál es el objetivo de la investigación a la cual se me ha invitado a participar?

Mucha gente tiene una infección bacteriana en el estómago por un organismo conocido como *Helicobacter pylori*. Esta bacteria puede causar enfermedad del estómago y duodeno, como úlcera, gastritis, duodenitis, cáncer gástrico, linfoma MALT de bajo grado de malignidad. Sin embargo, no todos los que están infectados se enferman.

El propósito de esta investigación es identificar la bacteria *Helicobacter pylori* y determinar su capacidad de causar lesiones y su comportamiento frente a los actuales esquemas de tratamiento

en pacientes con síntomas digestivos. Al mismo tiempo se evaluará si algunos factores relacionados al individuo que está infectado por esta bacteria, como edad, sexo, factores genéticos, ascendencia mapuche, nivel socioeconómico y algunos hábitos de vida, se pueden asociar con la presentación clínica de enfermedades gástricas.

2. ¿Quiénes realizarán esta investigación?

La investigación será desarrollada por un grupo de investigadores de la Universidad de La Frontera, del Hospital Hernán Henríquez Aravena y de la Clínica Alemana de Temuco, quienes estarán liderados por la Dra. Mónica Pavez Aguilar y la Sra. Claudia Troncoso Muñoz, investigadoras principales de los proyectos (Universidad de La Frontera).

3. ¿Por qué he sido seleccionado para participar en esta investigación?

Para realizar esta investigación, es fundamental contar con la participación de pacientes que vayan a ser sometidos a una endoscopía digestiva alta, ya que independiente de su cuadro clínico, existe una alta probabilidad de encontrar en su muestra a la bacteria *Helicobacter pylori,* objeto de nuestro estudio.

4. ¿En qué consiste mi participación?

Si acepta participar de esta investigación, miembros del estudio le harán una encuesta para registrar antecedentes personales y clínicos relevantes, como edad, sexo, enfermedades que le hayan sido diagnosticadas con anterioridad y otras preguntas para identificar factores de riesgo que pudiera presentar. Igualmente, integrantes del equipo le realizarán algunas preguntas para determinar hábitos de vida como consumo de alcohol y tabaco y de consumo alimentario.

Según indicación de su médico tratante, se le realizará una endoscopia digestiva alta. Este procedimiento ya incluye la toma de muestras de tejido del estómago (biopsia) para una determinación rápida de *H. pylori* (test de ureasa) y el estudio de lesiones, adicionalmente le solicitamos, si usted decide participar en este estudio, la toma de dos muestras adicionales del mismo tamaño con fines de investigación. Cabe destacar, que dichas determinaciones no tendrán costo adicional para usted. A la vez, se le solicita su autorización para revisar información necesaria, desde su ficha clínica y nos permita luego cruzar estas informaciones con la finalidad de concretar el estudio.

5. ¿Puedo no participar de la investigación?

Usted puede decidir libremente su participación. El que no participe de nuestro estudio no tendrá ninguna consecuencia negativa para usted.

6. Si acepto participar, ¿Puedo arrepentirme después de haber comenzado el estudio?

Si, usted podrá comunicar su decisión de no continuar participando en cualquier momento de la investigación. Para ello, debe dirigirse a las investigadoras principales Dra. Mónica Pavez A. y Sra. Claudia Troncos Muñoz, quien realizará el proceso de eliminación del material biológico correspondiente a sus muestras. Vea datos de contacto más abajo en este documento.

7. ¿Qué riesgos podré experimentar en este estudio y como podrán ser minimizados?

Los riesgos asociados al procedimiento de endoscopia digestiva alta son independientes de si participa o no de la investigación y le serán informados por el personal médico a cargo.

Las biopsias del estómago le tomarán unos minutos al doctor y no le causará dolor. En ocasiones puede haber un pequeño sangrado en el sitio donde se toma la biopsia. Sin embargo, el sangrado es muy escaso y no tendrá ninguna consecuencia futura para usted. Es importante recalcar que la endoscopia con las tomas de muestras de rutina para el procedimiento endoscópico se realizará aun cuando usted decida no participar del estudio, por lo que la toma de muestras adicionales al estudio no genera ningún riesgo adicional para usted.

8. ¿Qué harán los investigadores con la información obtenida?

Todos los datos son absolutamente confidenciales. A los tubos que transportarán su muestra se les asignará un código. Los datos obtenidos se analizarán de manera grupal, sin identificar a quien corresponde cada medición. Los hallazgos de esta investigación serán divulgados a través de publicaciones científicas y podrán ser utilizados por el Estado para fines públicos, sin hacer mención personal a su participación en el estudio. Sin embargo, usted podrá pedir la información relacionada a su muestra en cualquier momento de la investigación. Para ello, podrá contactarse con la investigadora responsable Dra. Mónica Pavez A. quien le hará llegar por escrito la solicitud de la información requerida por usted.

9. ¿Qué beneficios obtendré después de participar en el estudio?

En el caso que su médico tratante le haya solicitado una endoscopía digestiva alta incluyendo una biopsia y test rápido de ureasa, nuestro estudio no le entregará información nueva que sea relevante para su diagnóstico, en los casos en que el resultado de este test sea negativo. En el caso

de que este ensayo dé un resultado positivo, se le entregará un informe con el resultado del test de susceptibilidad, si éste es solicitado por el paciente, el cual podrá ser compartido con su médico tratante para la evaluación de su tratamiento.

La mayor parte de los resultados de esta investigación no reportarán un beneficio directo para usted, sin embargo, el análisis global de los datos podrá contribuir en el conocimiento de los factores de riesgo que conllevan a enfermedad del estómago, lo cual podrá beneficiar a un gran número de personas.

10. ¿Recibiré dinero por participar?

No. Su participación es totalmente voluntaria y no remunerada.

11. ¿Con quién me puedo comunicar para resolver dudas u obtener información sobre los resultados de la investigación?

Usted puede comunicarse con los investigadores principales del estudio, la Dra. Mónica Pavez A. y la Sra. Claudia Troncoso Muñoz quienes lo podrá atender en horario de 9:00 a 17:00 hrs. en dependencias de la Universidad de La Frontera, en Av. Alemania 0458, cuarto piso, Laboratorio de Biología Molecular Aplicada. También puede contactarse al teléfono +56 452592802 o al correo electrónico monica.pavez@ufrontera.cl, troncosomunozc@gmail.com.

12. ¿Quién guardará este consentimiento informado?

Le entregaremos una copia a usted. Otra copia será archivada en el Laboratorio de Biología Molecular Aplicada, Centro de Medicina Traslacional de la Universidad de La Frontera.

13. ¿Qué sucederá con las muestras de biopsia una vez finalizada la investigación?

Tanto durante como después del estudio, las muestras y datos podrán ser almacenados en el Laboratorio de Biología Molecular Aplicada, Centro de Biomedicina - Universidad de La Frontera (Av. Alemania Nº0458) por un periodo no superior a 5 años, siendo la investigadora responsable (Dra. Mónica Pavez A.) la encargada de su almacenamiento. Estas muestras no serán utilizadas con fines comerciales, solamente podrán ser usadas en estudios futuros relacionados a los objetivos de este proyecto, siempre que usted lo autorice.

14. Si tengo dudas de mis derechos como participante del estudio, ¿a quién puedo dirigirme?

Usted puede dirigirse en cualquier momento a la encargada de este estudio, Dra. Mónica Pavez A., cuyos datos de contacto fueron detallados anteriormente en este documento. Así mismo, cualquier reclamo, comentarios o preocupaciones relacionadas con esta investigación o preguntas sobre sus derechos al participar en el estudio, Usted puede dirigirse al Presidente del Comité Ético Científico de la Universidad de La Frontera, Dr. Milko Jorquera, Fono: +56 - 452734114, email: cec@ufrontera.cl, o concurrir personalmente a las oficinas del Comité ubicadas en calle Av. Francisco Salazar Nº01145, Temuco, Pabellón B, 1°Piso, Sector Vicerrectorías en horario de 09:00 a 13:00 hrs – 14:30 a 17:00 hrs.

S4.2. Acta of the informed consent and certificate of approval by the Scientific Ethics Committee of the Universidad de La Frontera, Chile (Protocol no. 028/18).

ACTA DE CONSENTIMIENTO INFORMADO

Declaro que he sido informado (a), y estoy de acuerdo con los objetivos y análisis que serán realizados en los estudios "**Evaluación del perfil de virulencia y resistencia a antibióticos en *Helicobacter pylori* como determinantes de la severidad de lesiones gástricas y eventual falla terapéutica**" y "**Asociación de la virulencia de cepas de *Helicobacter pylori* con la severidad de las lesiones gástricas en modelos in vivo e in vitro. Papel de la progranulina en la evolución de las lesiones**" en los cuales estoy participando voluntariamente y que son ejecutados por investigadores de la Universidad de La Frontera, Clínica Alemana de Temuco y Hospital Hernán Henrique Aravena.

Además, conozco en que consiste mi participación en él. En relación con ello, acepto:

- Dar respuesta a un cuestionario y autorizar a los investigadores participantes el acceso a revisión de la ficha clínica para cruzar la información necesaria según los objetivos del estudio.

Si No

- Que se me tomen dos biopsias de mucosa gástrica destinada a cultivo *in vitro* para la búsqueda de la bacteria *H. pylori* y estudios genéticos asociados, como parte del procedimiento endoscopía digestiva alta solicitada por mi médico tratante.

Si	No

- El almacenamiento y utilización de las muestras obtenidas del procedimiento de endoscopia para las determinaciones propuestas en los objetivos planteados y en estudios futuros relacionados a estos proyectos por un periodo no superior a cinco años.

Si	No

-Que el personal encargado de la investigación se contacté en el futuro, para dar el consentimiento a nuevos estudios.

Si	No

En relación con los resultados del estudio, se me asegura que serán realizados de forma confidencial y se me comunicarán de forma escrita por el investigador a cargo, en caso de que yo los solicite. También se me ha informado de los alcances y/o beneficios que puedan obtenerse una vez finalizado el trabajo. Los resultados obtenidos podrán ser utilizados para una publicación científica guardando estricta confidencialidad.

Comprendo que la presente investigación no tendrá costo monetario o similar para mi persona y que puedo abandonar la investigación en el momento que yo lo estime conveniente, sin acarrear ningún tipo de perjuicio. Doy fe de que los procedimientos del estudio han sido explicados a plenitud por el personal en terreno Sr/Sra…………………………………………………………………………………….... También se me han informado detalladamente mis derechos como participante del estudio.

………………………………………………………...   ……………………

…………………………

Nombre participante   Firma

Fecha

…………………………………………………………….   ……………………

…………………………

Nombre Investigador   Firma

Fecha

………..…………………………………………………..   ……………………

…………………………

Director del establecimiento   Firma

Fecha

UNIVERSIDAD DE LA FRONTERA

COMITÉ ÉTICO CIENTÍFICO

ACTA N°028_18

ACTA DE EVALUACIÓN
PROTOCOLO DE INVESTIGACIÓN FOLIO N° 111_17

En Temuco, a 14 de mayo de 2018, el Comité Ético Científico de la Universidad de La Frontera, mediante procedimiento de revisión de evaluación expedita, Art N°46 del reglamento, estudio con enmiendas previamente aprobado (Resolución Exenta N°4183 de fecha 17 de octubre de 2017), ha evaluado y sancionado el Proyecto de Investigación, presentado para optar al Grado Académico de Doctor en Ciencias Mención Biología Celular y Molecular Aplicada, según se indica:

**Título**	**"Asociación de la virulencia de cepas de *Helicobacter pylori* con la severidad de las lesiones gástricas en modelos *in vivo* e *in vitro*. Papel de la progranulina en la evolución de las lesiones"**
**Investigador responsable**	Srta. Claudia Troncoso Muñoz<br>Alumna del Programa de Doctorado en Ciencias mención Biología Celular y Molecular Aplicada
**Prof. Guía**	Dra. Leticia Barrientos
**Tipo de Proyecto**	Tesis Doctorado
**Institución**	Universidad de La Frontera
**N° Folio del Proyecto UFRO**	111_17
**Decisión**	APROBADO
**Fecha de Presentación al Comité Ético Científico (CEC)**	27.11.2018
**Fecha enmiendas solicitadas por CEC a IR**	Entrevista 10.04.2018 V2<br>Memorándum N°089_18 27.04.2018 V3
**Fecha Respuesta Investigador Responsable a CEC**	24.04.2018 V2<br>04.05.2018 V3
**Versión**	N°3

Documentos Revisados CEC

- Carta de solicitud revisión de Proyecto de Investigación.
- Carta de Apoyo Decano, Director de Depto. o Instituto.
- Carta de apoyo del encargado de laboratorio.
- Versión completa y definitiva del Proyecto de Investigación.
- Resumen del Proyecto.
- Modelo de Consentimiento Informado
- Formulario solicitud revisión investigación en Seres Humanos y Bioseguridad.
- Curriculum IR
- Curriculum Prof. Guías.
- Certificado Examen de Calificación.

Universidad de La Frontera, Comité Ético Científico, Av. Francisco Salazar N° 01145, Fono: 45/2734114, Temuco
